# Dental anxiety and dental attendance among 25-year-olds in Norway: time trends from 1997 to 2007

**DOI:** 10.1186/1472-6831-11-10

**Published:** 2011-03-22

**Authors:** Anne N Åstrøm, Erik Skaret, Ola Haugejorden

**Affiliations:** 1 Faculty of Medicine and Dentistry, University of Bergen, N-5020, Bergen, Norway; 2 Faculty of Dentistry, University of Oslo, Blindern, N-0317 Oslo, Norway

**Keywords:** dental anxiety, dental attendance, time trends

## Abstract

**Background:**

So far, there are few studies considering the development of dental anxiety and dental attendance patterns across time in the general population of Norwegian adults. This study aimed to 1) determine the frequency of dental anxiety and regular dental attendance among 25-year-olds in Norway in 1997 and 2007, 2) to study the development (time trend) of dental anxiety and the socio-behavioral distribution of dental anxiety from 1997 to 2007.

**Method:**

Random samples of 1,190 and 8,000 25-yr-olds were drawn from the populations of three counties in Western Norway in 1997 and 2007, respectively. The eligible participants received questionnaires by mail including questions on socio-demographics, dental anxiety (DAS) and dental attendance.

**Results:**

In 1997, 11.5% males versus 23% females reported high dental anxiety (DAS ≥ 13). Corresponding figures in 2007 were 11.3% and 19.8%. The proportions who had attended yearly for a dental check-up during the past 5 years fell from 62% in 1997 (men 56.9% and women 66.4%) to 44.6% (men 38.1% and women 48.6%) in 2007. After controlling for potential confounding factors, the 25-year-olds were 1.4 times more likely to report dental anxiety in 1997 compared to 2007. The decrease was largely attributable to a lower mean DAS score among higher educated females in 2007 than in 1997. The discrepancy in dental anxiety between regular and non-regular dental attendees had decreased, largely attributable to a decline in dental anxiety among irregular dental attendees.

**Conclusion:**

The study showed reduced dental anxiety and dental attendance among 25 year-olds in Norway from 1997 to 2007. This study points to the importance of controlling for possible changes in socio-demographic distributions when different cohorts are compared.

## Background

Dental anxiety is a serious problem affecting a significant part of the population. The prevalence of dental anxiety has been explored in a variety of populations and cultures during the last 30 years [1,2]. A range of socio-demographic, behavioral and psychosocial factors have been related to dental anxiety [3-5]. Dental anxiety is one of several factors related to whether or not people attend dental care [6]. The relationship between dental anxiety and irregular dental attendance and avoidance of dental care is well established [7-10], and also the differences by gender and age, with higher prevalence of dental anxiety in women than in men [2]. Dental attendance is influenced by socioeconomic variables [11-13], while the relationship between those factors and dental anxiety is more unclear [4].

Smith et al. [1] suggested stability in dental anxiety scores over time, based on a literature review covering the last 50 years. They found neither a significant increase nor decrease in self-reported anxiety levels among US college students [1]. The results from a great number of prevalence studies over time have indicated a similar situation in Europe, but few have presented time trends for dental anxiety and related behaviors based on comparable samples [14-17]. Even if the age of onset of dental anxiety seems to be about 12 years [18], some studies indicate that younger adults are particularly vulnerable to onset, and that dental anxiety may also arise during adolescence and adulthood [16,19]. A recent review, considering children and adolescents from 15 different populations has estimated a prevalence of dental anxiety of 9% (range 5.7% - 19.5%) [2]. In a cohort study, Thomson et al. [2] found an increase in dental anxiety from 15-26 years of age [17]. A longitudinal study of women in Sweden found that dental anxiety declined from middle- into older ages [20]. Humphris and King [21] compared the prevalence of high dental anxiety across past distressing experiences and found a prevalence of high dental anxiety amounting to 11.2% and past distressing experiences being a significant correlate among UK university students. Regional Norwegian studies have indicated that the prevalence scores among adolescents have reached the prevalence in the adult population [22,23]. These results suggest that the prevalence of dental anxiety is most stable in young adults, making this a suitable age group for exploring time trends for dental anxiety and related behaviors.

Using Aday and Andersen's framework for the study of access to medical care [24], Scheutz and Heidmann [6] explored determinants of utilization of dental care among 20-34-year olds. They found that in Denmark the following factors were associated with irregular use of dental services: age, sex, exercise habits, costs, dental anxiety, and perceived own dental health. A comprehensive review by Pavi et al. [12] presented 18 studies reporting the association between dental visiting patterns and social and economic indicators, and confirmed a stable relationship independent of instruments used for the measurements of socio-economic status and dental attendance. They also surveyed two populations in different social environments in Scotland and showed that social environment (affluent vs. deprived) was the strongest predictor of dental attendance, and that dental anxiety was negatively associated with attendance. Based on data from the 1998 Adult Dental Health Survey in UK, Donaldson et al. [13] concluded that barriers to regular dental attendance such as dental anxiety might mediate the relationship between socio-economic status and number of sound teeth. Using data from a complete cohort study, Crocombe et al [25], showed that individuals growing up with low childhood socioeconomic status were less likely to adopt stable dental attendance patterns as adolescent and young adult adults than were their counterparts growing up with high SES.

In the UK, the age group 16-24 years has been reported to attend dentists less frequently than they did five years ago [26]. In Norway Holst et al. [27] found that the proportions of adults (21 years or older) who had visited the dentist during the last year and during the two last years were 78% and 87%, respectively. For the age group 21-29 years, 76% had been to the dentist during the last two years. Støle et al. [28] found that in 1983 54% of 23-24-year-olds had been to the dentist yearly, and 87% had visited the dentist during the last two years. Corresponding figures for this age group in 1994 were 66% and 85%, respectively. For individuals who had not been to the dentist during the last year, 18% (1983) vs. 17% (1994) reported dental anxiety as the major reason. According to reports from Official Statistics of Norway [29], the total number of people examined/treated by public dentists decreased from 2005 to 2006, especially among children and adolescents in the age groups 3-18 and 19-20 years. It seems logical to assume that an increased attention towards the problem of dental anxiety and patient comfort (based on new technology) will influence the incidence of dentally anxious- and avoidant patients in the population. So far, there is a lack of studies considering the development of dental anxiety and dental attendance patterns across time among young adults in the general populations.

### Purpose

Targeting cohorts of 25-year-olds in South-West Norway, the aims of the present study were 1) to determine the frequency of dental anxiety and dental attendance in 1997 and 2007 and 2) to study the development (time trend) of dental anxiety and the socio-demographic and behavioral distribution of dental anxiety from 1997 to 2007.

## Methods

Simple random samples, (using individuals as the primary sampling unit) of 1,190 and 8,000 25-year-old residents (born in 1972 and 1982) were drawn from the populations of three counties in Western Norway in February 1997 and January-February 2007, respectively. The participants were informed that participation was voluntary and return of the completed questionnaire was considered as the informed consent. Permission to carry out the studies was granted by the Privacy Ombudsmann for all Norwegian universities and separately from the University of Bergen. The eligible participants received structured questionnaires by mail with an introductory letter explaining the purpose of the study and a promise of a reward in an attempt to improve participation. After a reminder, completed questionnaires were received from 736 (response rate 62%) and 1,509 (response rate 19%) 25-year-olds in 1997 and 2007, respectively. Of the subjects who replied in 1997, 51% were women and 38.7% reported higher level of education (i.e. more than 13 years). The corresponding figures in 2007 were 63% and 46.5%. According to the Central Bureau of Statistics of Norway (CBS), the distribution of respondents deviated only slightly from the corresponding population statistics in 1997. In 2007, the sample figures differed from the corresponding population statistics as shown in Table 1. In 2005, 21.3% of the population of 25-year-olds had an annual income of 400.000 NOK or more. The corresponding proportion in the 2007 sample was 29.6%. In 2006, 34.9% of the population versus 46.5% of the 2007 respondents had at least 13 years (higher) education. In 2007, females constituted 63.3% of the sample whereas the corresponding population parameter was 49.9% (Table 1).

**Table 1:** Annual income, educational level and gender in the sample of 25-year old and in the total population of 25-year-olds in Western Norway in 2005, 2006 and 2007

Variables	2007 respondents %	2005,2006,2007 population according to CBS % (year)
Annual income: 400.000 NOK or higher	29.6	21.3 (2005)
Educational level: high (> 13 yr)	46.5	34.9 (2006)
Gender: females	63.3	49.9 (2007)

### Instruments

#### Independent variables

The structured questionnaires covered aspects of socio-economic status and oral health related behaviors. The following socio-demographic factors were assessed in 1997 and 2007: gender [(1) male and (2) female] and educational level [(1) lower level (13 year or less), (2) higher level (> 13 years)]. Dental attendance during the past 5 years were recorded as (1) yes, annually (2) no, less than annually. Annual income was assessed as (1) low < 400.000NOK per yr and (2) high ≥ 400.000NOK per yr.

#### Dependent variables

The Dental Anxiety Scale (DAS) [30] used to measure dental anxiety, is psychometrically evaluated for initial screening of extreme dental fear [31], also in Norway [32,33]. Four items rated on a 5-point Likert scale yield sum scores from 4 to 20. Missing data on the DAS items were substituted with the mean score of the rest of the items. Missing 20% or more of the items was the criterion for not having a DAS sum-score. Internal consistency of DAS was assessed by use of Cronbach's alpha and was found to be 0.91 in 1997 [32] and 0.90 in 2007. High dental anxiety was defined as DAS ≥ 13.

### Statistical analyses

Data were analyzed using SPSS version 15.0 (Chicago, IL, USA). Categorical data were analyzed using cross-tabulation with chi-square statistics. The difference in response rate between males and female subjects in 2007 (Tables 1 and 2) compared with the population of 25-year-olds and the respondents in 1997 necessitated adjustment (direct standardization) of overall frequency rates and mean anxiety scores for comparative purposes. The number of male and female respondents in the three counties on 1^st ^January 2007 was used as the standard population. Determinants of dental anxiety and dental attendance were assessed using multiple logistic regression analysis with odds ratio (OR) and 95% confidence intervals (CI). The level of significance was set at 5%.

**Table 2:** Frequency distribution of socio-demographics and dental attendance and their categories by survey year

Independent variables	Categories	1997 % (*n*)	2007 % (*n*)
Gender	Male (1)	48.3 (348)	36.7 (548)**
	Female (2)	51.7 (373)	63.3 (947)
Dental attendance	Once a year (1)	62,0 (448)	44.6 (650)**
	Less than once a year (2)	38.0 (275)	55.4 (808)
Education	Lower - ≤ 13 yr(1)	61.3 (417)	53.5 (762)
	High- > 13 yr (2)	38.7 (263)	46.5 (663)**
Annual income	< 400.000NOK		70.4 (958)
	≥ 400.000 NOK		29.6 (402)

**p < 0.001

## Results

### Distribution of 25-year-olds by socio-demographics and dental attendance in 1997 and 2007

Table 2 depicts the percentage distribution of participants according to socio-demographic characteristics and dental attendance behavior by survey year. The gender distribution and educational level differed statistically significantly between survey years; with more female- and higher educated participants in 2007 than in 1997. The proportions who had attended yearly for a dental check-up during the past 5 years were 62% (adjusted: 61.9%, *n *= 718) and 44.6% (adjusted: 43.3%, *n *= 1 449) in 1997 and 2007, respectively (*p *< 0.001). The corresponding sex-specific rates were 56.9% for men and 66.4% for women (1997), 38.1% for men and 48.6% for women (2007).

### Frequency and socio-demographic distribution of dental anxiety in 1997 and 2007

Mean DAS scores according to survey year were 8.7 (sd 3.7) (male: 7.9, sd 3.3, female: 9.1, sd 3.9) (adjusted mean 8.5) and 9.0 (sd 4.0) (male: 8.1, sd 3.5, female 9.5, sd 4.2) (adjusted mean: 8.8) in 2007 and 1997, respectively. In 1997 and 2007 17.5% (adjusted: 17.2%) and 16.7% (adjusted: 15.5%) scored high on the dental anxiety scale. As reported in Table 3, dental anxiety varied systematically with gender and dental attendance patterns within each survey year. In 1997, 11.5% males versus 23.0% females reported high dental anxiety (DAS ≥ 13). Corresponding figures in 2007 were 11.3% and 19.8%. A total of 29.1% less frequent- versus 11.4% (*p *< 0.001) frequent dental attendees reported high dental anxiety in 1997. The corresponding figures in 2007 were 21% and 13.6%. Dental anxiety varied systematically with educational level, with higher educated reporting dental anxiety more- and less frequently than their lower educated counterparts in 1997 and 2007, respectively (Table 3).

**Table 3:** Percentage distribution of respondents according to dental anxiety, gender, dental attendance patterns and survey year

	1997			2007		
	Total	Male	Female	Total	Male	Female
High dental anxiety	17.5 (125)	11.5 (40)	23.0 (85)**	16.7 (246)	11.3 (61)	19.8 (185)**
		Dental attendance (Low) ^a^	Dental attendance (High)^b^		Dental attendance (Low)^a^	Dental attendance (High)^b^
		29.1 (59)	11.4 (51)**		21.0 (99)	13.6 (88)*
		Education-Low ^c^	Education-High ^d^		Education-Low ^c^	Education-High ^d^
		15.8 (65)	21.0 (54)*		18.9 (143)	13.9 (92)

Percentages of those who had high dental anxiety (DAS ≥ 13).

**p < 0.001

*p < 0.05

^a^- dental care less than once a year

^b ^dental care once a year

^c ^≤ 13 yr

^d ^> 13 yr

### Changes in dental anxiety and its socio-demographic distribution between 1997 and 2007

Logistic regression analysis with 1997 and 2007 samples combined, controlling for gender, educational level, dental attendance and two-way interaction terms revealed that compared to males and irregular dental attendees, females and regular dental visitors were respectively 2.4 times more- and 0.4 times less likely to report high dental anxiety (Table 4). As compared to 2007, the odds ratio for having dental anxiety in 1997 was 1.4 (95% CI 1.0-1.9). Significant second order effects (two-way interactions) were observed for survey year × dental attendance and for survey year × educational level. Probing the results in each survey year highlighted the direction of these interactions. As compared to irregular dental attendees, regular dental attendees were 0.2 times and 0.5 times less likely to have dental anxiety in 1997 and 2007, respectively. In 2007, higher educated were less likely than lower educated to have dental anxiety (OR 0.6, 95% CI 0.3-0.9). In 1997, higher educated were more likely than lower educated to have dental anxiety (OR 2.2 95% CI 1.2-3.8).

**Table 4:** Adjusted odds ratios (OR) and 95% confidence interval (CI) of having dental anxiety according to year of survey, gender, and dental attendance behavior (included in analysis *n *= 1672)

Variables	OR	95% CI
Year: 2007	1	
1997	1.4	1.0-1.9
Gender: Male	1	
Female	2.4	1.6-3.4
Education: ≤ 13 yr	1	
> 13 yr	0.9	0.6-1.3
Dental attendance: less than once a year	1	
: once a year	0.4	0.3-0.5

** *p *< 0.001

## Discussion

This study explored possible time trends in the frequency of dental anxiety and dental attendance behavior among 25-year-olds in Western Norway from 1997 to 2007. The cross-sectional studies in 1997 and 2007 revealed a decrease in the frequency of dental anxiety from 17.6% to 16.7% and a decrease in the frequency of annual dental attendance from 62% to 44.6% among 25-year-olds. Whether those findings should be interpreted as period effects (i.e. events that have taken place between 1997 and 2007) or cohort effects (i.e. due to historical differences of different cohorts) cannot be inferred from the present empirical analysis. Random samples from the general population of 25-yr-olds and standardized survey instruments made it possible to describe changes in dental anxiety and dental attendance across the 10 year period. However, some methodological limitations should be considered when interpreting the results. The moderate (62%) and low (19%) response rates achieved in 1997 and 2007, may have biased the estimates and thus constitutes a threat to the external validity of the results. Unfortunately, lack of information about non-responders made a detailed comparison with responders on relevant background variables impossible in either survey year. Nevertheless, comparing the 2007 group of respondents with population statistics revealed an overrepresentation of females and subjects with higher education (Table 1). Owing to the fact that female gender is associated with more- and higher education with less dental anxiety, it is difficult to assume any over- or underestimation of the real dental anxiety prevalence in 2007. The decrease in dental anxiety across time, as observed in this article, might nevertheless be attributed to selection bias with overrepresentation of highly educated healthy participants in 2007. As indicated in Table 2, the study populations of 1997 and 2007 differed with respect to socio-demographic characteristics that are of relevance for dental anxiety, indicating substantial differences in the demographic composition of the two cohorts investigated. To minimize biasing effects on time lag differences regarding dental anxiety, sex standardization of the crude prevalence estimates of dental attendance and dental anxiety was performed in addition to multiple logistic regression analyses considering the potential confounding variables.

Dental anxiety varied systematically with gender, dental attendance pattern and educational level within each survey year. In 1997, 11.5% males versus 23.0% females reported high dental anxiety (DAS ≥ 13). Corresponding figures in 2007 were 11.3% and 19.8%, respectively. The higher prevalence of dental anxiety among women compared to men is in accordance with a number of previous studies in Norway and elsewhere [23,32,34-36]. This finding is consistent with a larger body of literature suggesting that females generally are more likely than males to be diagnosed with anxiety and phobia related problems [18,37].

As depicted in Table 3, dental anxiety did not vary systematically with survey year in the bivariate analyses. However, controlling for possible confounding variables (i.e. socio-demographic differences between the study groups in 1997 and 2007) revealed a statistically significant decrease in dental anxiety from 1997 to 2007; the 25-year-olds were 1.4 times more likely to report dental anxiety in 1997 compared to 2007. Moreover, according to significant second order effects (two-way interactions), the discrepancy in dental anxiety between regular and non-regular dental attendees declined across time. This reduced inequality in dental anxiety was largely attributable to the decline in dental anxiety observed among irregular dental attendees. The association between dental anxiety and educational level was unstable across time, with the frequency of dental anxiety being highest and lowest among subjects with higher education in 1997 and 2007, respectively and indicating a decrease among the higher educated subjects only, from 21.0% in 1997 to 13.0% in 2007. Further analyses revealed that the decrease in dental anxiety from 1997 to 2007 was largely attributable to a lower mean DAS score among higher educated females in 2007 than in 1997. Changes in anxiety levels among males and females with a low educational level were not statistically significant. This result indicate that the relationship between dental anxiety and socio-demographics is still unclear, even though some studies have shown that dental anxiety is most frequent in lower socio-economic status groups [5,38].

The proportion who had attended yearly for a dental check-up during the past 5 years declined from 62% in 1997 to 44.6% in 2007. The reduction in dental visiting pattern confirms the tendency observed in previous Norwegian studies [27-29]. Consistent with numerous studies in the field of dental anxiety, subjects who used dental care less than once a year (irregular dental attendees) reported dental anxiety more frequently than subjects visiting dentists annually (regular dental attendees) [4,6,8-10,25,32]. To the extent that people of lower socio-economic status lack money and other resources, they are expected to visit dental health care services less frequently. These individuals are caught in a vicious circle where the irregular dental attendance pattern leads to dental problems and a symptomatic visiting pattern; with an increased risk of unpleasant treatment experiences and increased dental anxiety [10]. Our finding in Norway revealed that it will be a challenge to improve the dentists' ability to bring these patients out of this vicious circle.

The reduced difference in dental anxiety between regular and irregular dental attendees observed from 1997 to 2007 suggests that reduction in dental attendance is influenced by factors other than dental anxiety. Norwegian adolescents have experienced that the public dental service has gradually extended the recall intervals [39], due to improved dental health [40,41] and increased focus on the high risk group approach. When adolescents gradually assume responsibility for their own dental visits, also subjects with low dental anxiety have learned that it is reasonably safe to increase the time intervals between their dental visits. A reduction in the frequency of dental attendance among 25-yr-olds during the last 10 years does not necessarily represent a problem behavior.

## Conclusion

This study found reduced dental anxiety and dental attendance among 25-year-olds from 1997 to 2007 in South-Western Norway. Moreover, reduced inequality in dental anxiety between regular and irregular dental attendees that was largely attributable to the decline in dental anxiety observed among irregular dental attendees. This study points to the importance of controlling for possible changes in socio-demographic distributions when different cohorts are compared. Lack of control of related variables would have made the interpretation of the results less reliable.

## Competing interests

The data was obtained from the Central Bureau of Statistics Norway (CBS), and made available in anonymous form by the Norwegian Social Science Data Services (NSD). The authors alone are responsible for the content and writing of the paper and report no conflicts of interest.

## Authors' contributions

ANÅ: principle investigator, conceived of the studies, designed the studies and, performed statistical analyses and manuscript writing. ES: contributed substantively to the work with statistical analyses of data and manuscript writing. OH: provided valuable comments to the paper in general and was actively involved with the data analysis and manuscript writing. All authors red and approved the final manuscript.

## Pre-publication history

The pre-publication history for this paper can be accessed here:

http://www.biomedcentral.com/1472-6831/11/10/prepub
